# Nuclear-Biased DUSP6 Expression is Associated with Cancer Spreading Including Brain Metastasis in Triple-Negative Breast Cancer

**DOI:** 10.3390/ijms20123080

**Published:** 2019-06-24

**Authors:** Fan Wu, Robert D. McCuaig, Christopher R. Sutton, Abel H. Y. Tan, Yoshni Jeelall, Elaine G. Bean, Jin Dai, Thiru Prasanna, Jacob Batham, Laeeq Malik, Desmond Yip, Jane E. Dahlstrom, Sudha Rao

**Affiliations:** 1 Melanie Swan Memorial Translational Centre, Faculty of Sci-Tech, University of Canberra, Bruce ACT 2617, Australia; Fan.Wu@canberra.edu.au (F.W.); Robert.McCuaig@canberra.edu.au (R.D.M.); Chris.Sutton@canberra.edu.au (C.R.S.); Abel.Tan@canberra.edu.au (A.H.Y.T.); Yoshni.Jeelall@canberra.edu.au (Y.J.); Jin.dai@canberra.edu.au (J.D.); Thiru.Prasanna@canberra.edu.au (T.P.); Jacob.batham@canberra.edu.au (J.B.); 2 Anatomical Pathology, ACT Pathology, The Canberra Hospital, Canberra health Services, Garran ACT 2606, Australia; Elaine.Bean@act.gov.au (E.G.B.); Jane.Dahlstrom@act.gov.au (J.E.D.); 3 Department of Medical Oncology, The Canberra Hospital, Canberra health Services, Garran ACT 2606, Australia; Laeeq.Malik@act.gov.au (L.M.); Desmond.Yip@anu.edu.au (D.Y.); 4 ANU Medical School, College of Health and Medicine, The Australian National University, Canberra ACT 0200, Australia

**Keywords:** circulating tumor cells (CTCs), DEPArray, dual-specificity phosphatase, HER2, brain metastasis, single cell analysis, triple-negative breast cancer (TNBC)

## Abstract

DUSP6 is a dual-specificity phosphatase (DUSP) involved in breast cancer progression, recurrence, and metastasis. DUSP6 is predominantly cytoplasmic in HER2+ primary breast cancer cells, but the expression and subcellular localization of DUSPs, especially DUSP6, in HER2-positive circulating tumor cells (CTCs) is unknown. Here we used the DEPArray system to identify and isolate CTCs from metastatic triple negative breast cancer (TNBC) patients and performed single-cell NanoString analysis to quantify cancer pathway gene expression in HER2-positive and HER2-negative CTC populations. All TNBC patients contained HER2-positive CTCs. HER2-positive CTCs were associated with increased ERK1/ERK2 expression, which are direct DUSP6 targets. DUSP6 protein expression was predominantly nuclear in breast CTCs and the brain metastases but not pleura or lung metastases of TNBC patients. Therefore, nuclear DUSP6 may play a role in the association with cancer spreading in TNBC patients, including brain metastasis.

## 1. Introduction

Breast cancer is the most common malignant disease in women worldwide. While there has been significant progress in breast cancer treatment over recent decades through new chemotherapies, hormone therapies, targeted therapies, and more recently immunotherapies, 25–40% of breast cancer patients still develop primary resistance and metastasis to eventually die from their disease [1,2]. Identification of predictive resistance biomarkers has so far remained elusive, especially in triple negative breast cancer (TNBC), a breast cancer molecular subtype with currently few treatment options and an often-aggressive disease course.

Epithelial to mesenchymal transition (EMT) is a biological program in which epithelial cells lose cell–cell junctions, apical–basal polarity, and acquire an invasive mesenchymal phenotype. EMT is induced via several interacting signaling pathways including the WNT, TGF-β, Notch, Hedgehog, PI3-kinase/AKT, PKC-theta, and mitogen-activated protein kinase (MAPK) pathways [3,4,5]. EMT is implicated in cancer initiation, progression, metastasis, resistance to conventional therapies, and recurrence by inducing cancer stem cells (CSCs), tumor-initiating cells (TICs), and circulating tumor cells (CTCs) [3,5,6]. 

Breast CTCs are defined as cells circulating in the blood that lack CD45 expression but express cytokeratins 7, 8, 18, and 19 [7,8]. Breast CTCs are associated with progressive cancer and a higher risk of recurrence and metastasis [6,9]. Both HER2-positive CTCs and HER2-negative CTCs have been detected in patients with HER2-negative primary tumors (e.g., TNBCs) after adjuvant chemotherapy for the management of their primary breast cancer [9,10,11,12]. HER2-positive CTCs are associated with progression following multiple courses of systemic therapy in HER2-negative metastatic breast cancer patients [13]. 

Dual-specificity phosphatases (DUSPs) are a family of proteins responsible for dephosphorylating threonine/serine and tyrosine residues on their substrates, mainly MAP kinases. Several DUSPs are implicated in breast cancer metastasis, for example, DUSP1, DUSP4, and DUSP6 [4,14]. DUSP1 and DUSP4 are predominantly expressed in the nucleus, while DUSP6 is predominantly cytoplasmic [4]. DUSP6 has an interdomain linker region that contains a binding motif of ERK1/2 and nuclear export signal (NES) [15]. NES mediated nuclear-cytoplasmic shuttling of DUSP6 undergoes a highly specific interaction with ERK1 and ERK2 at dual threonine and tyrosine residues of the Thr-Glu-Tyr (TEY) motif, thereby inactivating DUSP6 [16]. Caspase-3 cleavage of DUSP6 mediates the subcellular localization and activation of ERK1/2 [15]. DUSP6 phosphorylation and ERK1/2 dephosphorylation creates a negative feedback loop to control ERK activity and indirectly the MAPK signal, which is vital for cellular proliferation and differentiation [15,16,17,18,19,20,21,22]. DUSP6 is enriched in HER2-positive breast cancer tumor [23], the overexpression of which confers resistance to tamoxifen endocrine therapy [4,24], and we previously showed that DUSP6 knockdown by RNA interference increased CSC formation but inhibited cell proliferation in TNBC cell lines in vitro [4,21]. DUSP6 is also upregulated in the cytoplasm of primary malignant breast cancer cells in HER2-positive breast cancer patients regardless of estrogen receptor/progesterone receptor (ER/PR) status [4]. Importantly, DUSP6 is cytoplasmic in ER^−^PR^−^HER2^+^ tissue but not in TNBCs [4]. The nuclear role of DUSP6 in EMT and metastasis in TNBC remains elusive. 

HER2-positive CTCs were recently identified in breast cancer patients with HER2-negative primary tumors [8]. However, it is unknown how the mRNA profiles of HER2-positive CTCs and HER2-negative CTCs differ. We therefore analyzed mRNA profiles in HER2-positive CTCs and HER2-negative CTCs isolated from five metastatic breast cancer patients with TNBC. At least one HER2-positive CTC was detected in every patient. mRNA expression profiles were distinct in HER2-positive and HER2-negative CTCs in the same patient. Pathway analysis revealed differential expression of genes involved in ERK1/2-MAPK signaling between HER2-positive and HER2-negative CTCs and involvement of DUSP6. We report for the first time that nuclear DUSP6 was present in CTCs from metastatic breast cancer patients regardless of CTC HER2 status. Importantly, nuclear-biased DUSP6 was also present in brain metastases but not pleura or lung metastases in TNBC patients, suggesting that the nuclear DUSP6-associated pathway participates in cancer spreading in TNBC, including brain metastasis.

## 2. Results

### 2.1. HER2-Positive and HER2-Negative CTCs Detected and Isolated from Primary TNBC Patients

**Figure 1:**
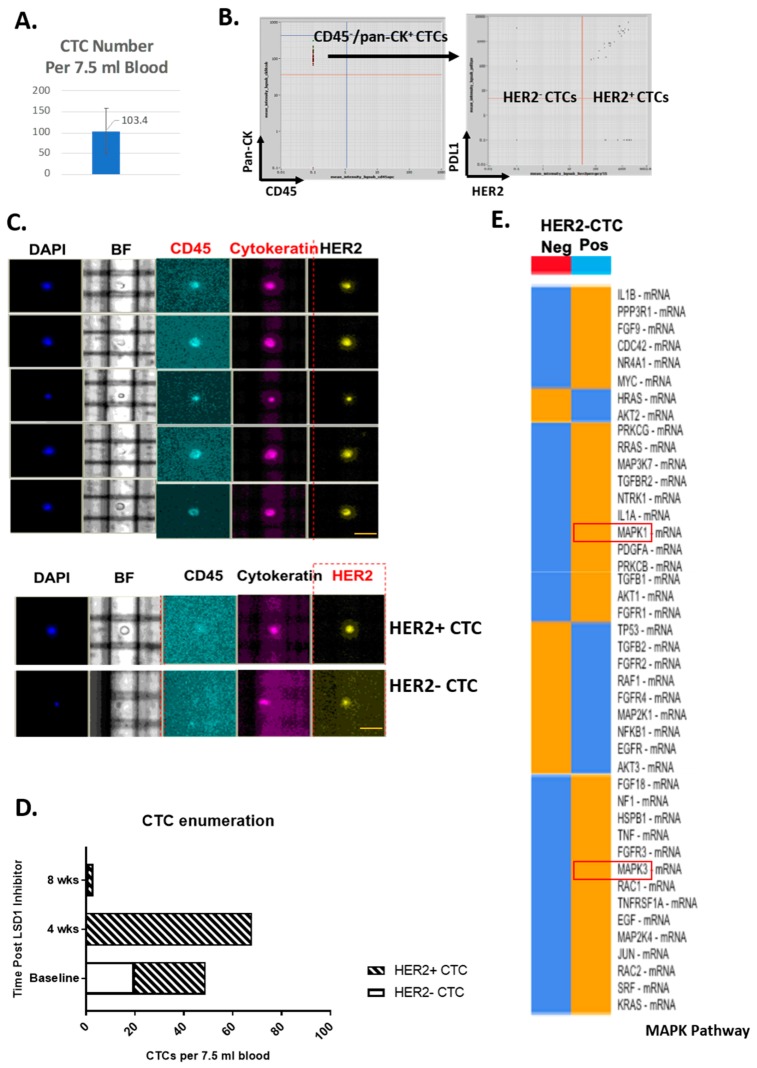
HER2-positive and negative circulating tumor cells (CTCs) have distinct mRNA profiles in metastatic triple negative breast cancer (TNBC) patients. CD45-negative enriched PBMCs were labelled with anti-human CD45-APC and pan-cytokeratin-FITC for DEPArray CTC enumeration and isolation. (**A**) CTCs were detected in metastatic TNBC patients. CTCs were enumerated in five metastatic TNBC patients using the DEPArray platform. Data represent the mean of CTC counts ± standard deviation in 7.5 mL equivalent whole blood. (**B**) Identification and isolation of HER2-positive and negative CTCs in the same patients. Plot shows the gating strategy for identifying HER2-positive and negative CTCs using DEPArray. Example images are indicated with scale bars (10 µm). (**C**) HER2-positive CTCs and HER2-negative CTCs using DEPArray. BF: brightfield. (**D**) CTC HER2 proportions over the course of chemotherapy in combination of Lysine-specific histone demethylase 1 (LSD1) inhibitor. Plot represents the number of HER2-positive and negative CTCs from 7.5 mL equivalent whole blood. (**E**) HER2-positive CTCs show distinct mRNA profiles in genes involved in mitogen-activated protein kinase (MAPK) signaling. NanoString analysis was performed using their pan-cancer progression panel to investigate differential gene expression in HER2-positive and negative CTCs isolated from the same patient. Data generated by pathway analysis using nSolver 4.0 software (NanoString). Orange indicates higher mRNA expression and blue indicates lower expression.

CD45^−^/pan-CK^+^ CTCs along with HER2-positive and HER2-negative CTC sub-populations were identified, enumerated and isolated from five metastatic TNBC patients using the DEPArray system (Silicon Biosystems, Castel Maggiore, Italy). There were 5 to 150 CTCs per 7.5 mL blood (in average of 103 CTCs per 7.5 mL blood) (Figure 1A,B). All TNBC patients in this group had HER2-positive CTCs detected ranging from 10% to 100% of total (Figure 1B–D). Post standard chemotherapy in combination with an inhibitor targeting Lysine-specific histone demethylase 1 (LSD1), HER2-positive CTCs were more frequent in patients with disease progression with increased total CTC number and less frequent in patients with stable disease (Figure 1D). 

### 2.2. Distinct ERK1/2-MAPK Pathway Gene Profiles in HER2-Positive CTCs and HER2-Negative CTCs

To assess the mRNA profiles of HER2-positive and negative CTCs, both populations were harvested from the same patient using the DEPArray system and, after cDNA synthesis and un-biased pre-amplification, expression of 739 mRNAs from the NanoString PanCancer Progression panel assessed. ERK1/ERK2-MAPK signaling pathway analysis showed that HER2-positive CTCs had decreased AKT1/2 mRNA expression and increased MAPK1 and MAPK3 expression, which are the direct substrates of DUSP6 (Figure 1E).

### 2.3. Nuclear Distribution of DUSP6 in CTCs and Brain Metastases from TNBC Patients

**Figure 2:**
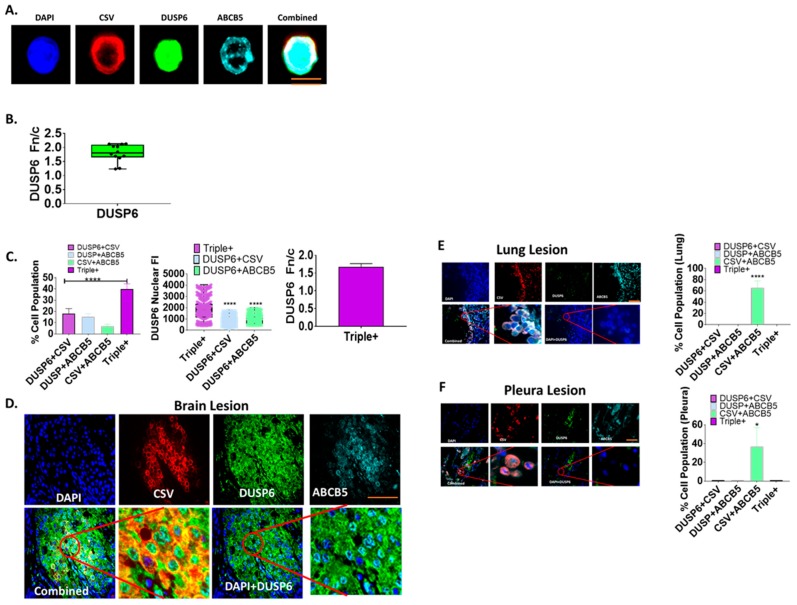
Nuclear-biased dual-specificity phosphatase 6 (DUSP6) in CTCs and brain metastases from metastatic TNBC patients. (**A**) Nuclear DUSP6 presented in CTCs from metastatic breast cancer patients. CTCs were labelled with anti-human DUSP6, cell surface vimentin (CSV), ABCB5, and DAPI for microscopy. (**B**) The nuclear bias of DUSP6 in CTCs from stage IV metastatic breast cancer patients (*n* = 5 patients). Nuclear/cytoplasmic intensity of DUSP6 was measured by microscopy. (**C**) DUSP6 expression in breast cancer metastases to the brain. Fluorescence labelling of DUSP6, CSV, and ABCB5 was performed for ASI microscopy analysis. Bar graph represents cells double-positive for either DUSP6/ABCB5/CSV or triple-positive for all three markers. The second bar graph displays the nuclear fluorescence intensity in double- or triple-positive cells for DUSP6. >500 cells counted per patient. Representative image of nuclear DUSP6 expression in (**D**) brain metastasis tissue (*n* = 21 patients). (**E**) DUSP6 expression in breast cancer metastases to the lung and (**F**) pleura (*n* = 5 patients) from metastatic TNBC patients. Bar graph represents cells double-positive for either DUSP6/ABCB5/CSV or triple-positive for all three markers. Scale bars = 10 µm and are shown in orange. Data represent mean ± SE. Unpaired *t*-test. *p < 0.05, ****p < 0.0001 denote significant differences.

Since DUSP6 directly dephosphorylates ERK1/ERK2, we reasoned that DUSP6 may participate in HER2-positive CTCs in metastatic breast cancer patients. DUSP6 showed nuclear and cytoplasmic expression and was biased towards nuclear expression in the CTCs as measured by the ratio of nuclear to cytoplasmic (Fn/c), (Figure 2A,B and Figure S1C). Moreover, nuclear DUSP6 was also co-expressed with cell surface vimentin (CSV) and ABCB5 known mesenchymal markers in CTCs (Figure 2A–C). We also examined the distribution of DUSP6 protein expression in brain metastasis tissue in TNBC patients (Figure 2C,D and Figure S1A,B). In 19 out of 21 patients, approximately 40% of cells were triple-positive for DUSP6, CSV, and ABCB5 (Figure 2C). Surprisingly, DUSP6 nuclear expression was also observed in the brain tissue, with highest DUSP6 expression observed in CSV^+^ABCB5^+^DUSP6^+^ triple-positive cells, (Figure 2C). To further understand whether nuclear expression of DUSP6 is exclusively presented in brain metastasis, we examined five lung and pleura metastases tissues from TNBC patients. Interestingly, virtually no neoplastic cell had nuclear DUSP6 expression in all five samples, although large numbers of CSV^+^ABCB5^+^ cells were detected (Figure 2E,F). 

### 2.4. Nuclear Distribution of DUSP6 in Primary Tumor Sections in the Chemotherapy or Immunotherapy Treated 4T1 Metastatic Mouse Model

**Figure 3:**
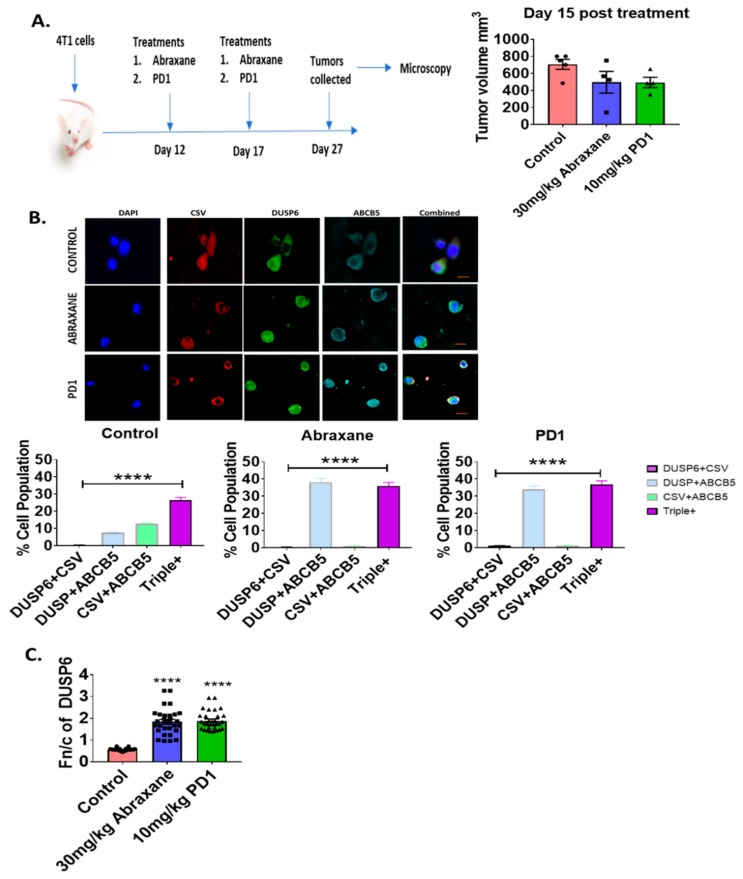
Nuclear-biased DUSP6 in the 4T1 metastatic mouse cancer model. (**A**) Treatment regimen using the BALB/c 4T1 breast cancer model and day 15 tumor volumes of mice treated with vehicle control, Abraxane, or PD-1 (*n* = 4). (**B**) DUSP6 expression in the primary 4T1 tumor model. Fluorescence labelling of DUSP6, CSV, and ABCB5 was performed for ASI microscopy analysis. Bar graph represents cells double-positive for either DUSP6/ABCB5/CSV or triple-positive for these three markers. *n* = 3 mice per group (>500 cells per patient counted). Data represent mean ± SE. Unpaired *t*-test. ****p < 0.0001 denote significant differences. Example images of each group are indicated with scale bars (10 µm). (**C**) DUSP6 Fn/c (ratio of nuclear to cytoplasmic staining) was quantified in the primary 4T1 tumor model. Fn/c was determined by performing ASI microcopy analysis and using ImageJ-Fiji to determine the ratio. Bar graphs represent fn/c mean ± SE. Unpaired *t*-test., ****p < 0.0001 denote significant differences. Example images are in 3B.

To understand whether nuclear DUSP6 is associated with drug resistance, we next examined DUSP6 expression in the 4T1 TNBC metastatic mouse breast cancer model treated with control vehicle, Abraxane (nab-paclitaxel, 30 mg/kg), or anti-PD1 (RMP1–14, 10 mg/kg) using the ASI digital pathology system. We first examined the changes in tumor volume caused by treatment with either Abraxane or anti-PD1 immunotherapy (Figure 3A) reduced overall tumor volume equally (Figure 3A). In surviving 4T1 tumor cells in Abraxane or anti-PD1-treated mice, nuclear DUSP6 was enriched in both triple-positive cells (CSV^+^ABCB5^+^DUSP6^+^) and the double-positive ABCB5^+^DUSP6^+^ tumor cell pool (Figure 3B). We next examined the Fn/c of DUSP6 expression that revealed that DUSP6 is predominantly cytoplasmic in control tumors but becomes nuclear biased in surviving tumor cells following treatment with Abraxane or anti-PD1 (Figure 3C). DUSP6 expression correlated with tumor cell survival in response to either chemotherapy (Abraxane) or immunotherapy (anti-PD1) (Figure S2).

### 2.5. Novel Inhibition of Nuclear DUSP6 Mediated the Expression of ABCB5 and CSV In Vitro

**Figure 4:**
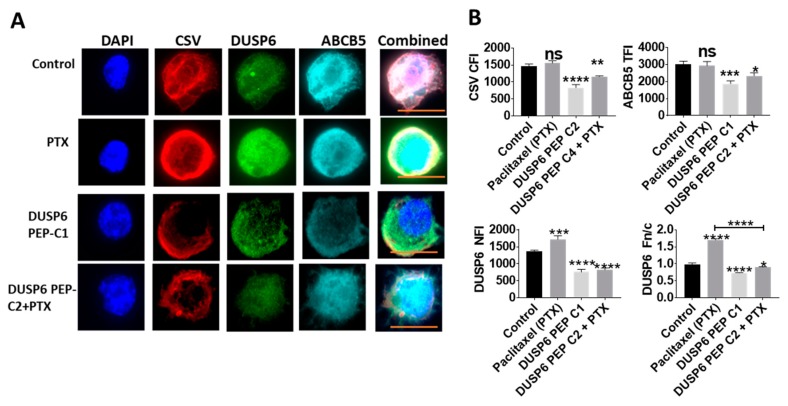
Inhibition of nuclear DUSP6 with novel peptide inhibitor. DUSP6, CSV and ABCB5 were labelled in TNBC cell line, MDA-MB-231 and treated with either vehicle alone, Paclitaxel (PTX) or DUSP6 Nuclear Peptide inhibitor (DUSP6 PEP, C1 = 60 mg/mL) or a combination (PTX + C2 40 mg/mL) with *n* > 20 individual cells. Representative images for each dataset are shown (**A**). Graphs represent the Fn/c, nuclear fluorescence intensity (NFI), cytoplasmic fluorescence intensity (CFI) or total fluorescence intensity (TFI) measured with the ASI automated digital pathology system (**B**). Scale bar is in orange and equals 10 μM. *p < 0.05, **p < 0.01, ***p < 0.001, ****p < 0.0001 denote significant differences. ns denotes non-significant.

To understand the effect of inhibition of nuclear DUSP6 in TNBC, we designed a novel competitive peptide inhibitor targeting a bipartite nuclear localization signal (NLS) of DUSP6 that was identified using the NLS Mapper predictive tool. In MDA-MB-231 TNBC cells, treatment of Paclitaxel induced nuclear translocation of DUSP6. In comparison, the DUSP6 NLS peptide inhibitor significantly reduced nuclear DUSP6 expression (Figure 4A) and inhibited the expression of CSV and ABCB5 in vitro suggesting alteration of protein function (Figure 4A,B). These results suggest that inhibition of nuclear expression of DUSP6 subsequently impacts expression of the mesenchymal, stem-like markers such as CSV or ABCB5.

### 2.6. Nuclear Association of DUSP6 with P300 and H3K9me2.

Nucleosomes are enriched in highly compacted chromatin structures which are transcriptionally silent. An important mechanism of regulating gene expression is chromatin remodeling which can be orchestrated via a number of mechanisms including post-translational modifications (PTMs) of histone proteins such as the repressive mark histone H3K9me2 as well co-activator proteins such as the histone acetyltransferase P300 that mediate active gene transcription and we have previously show to interact with DUSP family members [4]. 

**Figure 5:**
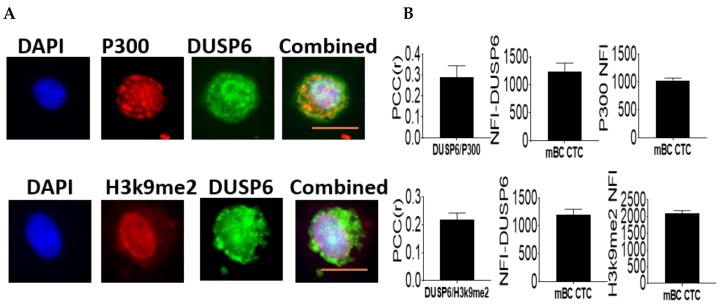
Nuclear DUSP6 interaction with P300 or H3k9me2. Confocal laser scanning microscopy was performed on CTCs (circulating tumor cells) isolated from metastatic breast cancer patients (Stage IV *n* = 10). Cells were fixed and probed with primary rabbit antibodies to DUSP6 and primary mouse antibodies to P300 or H3k9me2 followed by the corresponding secondary antibody conjugated to Alexa-Fluor 568 or Alexa-Fluor 488. Representative images for each antibody dataset pair are shown: red = p300 or H3k9me2; green = DUSP6. (**A**) The The Pearson correlation coefficient (PCC) was determined as described in methods. PCC indicates the strength of relation between the two fluorochrome signals for at least 20 individual cells ± SE. Bar graphs (**B**) indicate the total NFI of DUSP6, p300, or H3K9me2 as measured using ASI software to select the nucleus of each cell and measure the total NFI signal minus background for at least 20 individual cells ± SE. Scale bar is in orange and equals 10 μM.

Our results of H3k9me2 and DUSP6 co-expression revealed that while there was some positive co-localization the score was low (Figure 5). Whereas analysis revealed that P300 positively co-localized with DUSP6 in the nucleus of these CTCs with a stronger positive score (Figure 5). Collectively our data suggest that DUSP6 associates with P300 akin to our previous results for DUSP4 [4]. This suggests a potential role for DUSP6 to mediate active gene transcription programs in CTCs from metastatic breast cancer patients.

## 3. Discussion

TNBC remains the most difficult group of metastatic breast cancers to manage due to a lack of targetable proteins expressed in these tumor cells [1]. Despite therapeutic advances, the overall response and survival rates for patients with TNBC are low using conventional chemotherapy and novel immunotherapy compared to other solid organ malignancies. Increased CTC numbers are associated with rapid cancer progression and a higher risk of recurrence and metastasis. Here we confirmed the presence of both HER2-positive and negative CTCs in patients with a primary tissue diagnosis of TNBC, supporting previous findings [9,11,12]. For the first time, we compared the mRNA profiles of these two populations directly in the same patient using a panel of cancer progression-related genes, and found differential MAPK pathway gene expression. Specifically, there was increased expression of *MAPK1/2*, encoding ERK1/2, which are the direct dephosphorylation targets of DUSP6, in HER2-positive CTCs. 

DUSP6 is predominately expressed in the cytoplasm of breast cancer cells such as the commonly used cell lines MCF-7 and MDA-MB-231 [4]. DUSP6 nuclear expression increases in tumor cells that have transformed into CSCs via EMT [4]. Dual inhibition of DUSP6 and DUSP1 significantly reduces CSC formation in vitro. We previously showed that DUSP6 was exclusively cytoplasmic in primary HER2^+^ER^−^PR^−^ breast cancers from metastatic breast cancer patients, with no nuclear DUSP6 expression in primary TNBCs [4]. Interestingly, in CTCs, DUSP6 was nuclear in all vimentin-positive mesenchymal CTCs from stage IV metastatic patients evaluated. In 19 out of 21 patients TNBC metastatic breast cancer patient brain metastases, DUSP6 had nuclear expression, suggesting that the brain metastasis may form from CTCs via a nuclear DUSP6-dependent pathway. In comparison, our previous study showed that TNBC patients had undetectable levels of nuclear DUSP6 expression [4]. Overall, this suggests that distinct mechanisms are likely at play in the aggressive cancer cells in primary tissue, lung and pleura metastases tissue compared to brain metastases events. Future studies, particularly larger blind studies, will be required to fully validate the results of this study.

These results have a number of potential important implications for patient care. First, a subset of TNBC patients may in fact benefit from trastuzumab (Herceptin) therapy if they harbor significant numbers of HER2-positive CTCs and that HER2-CTC testing may be required in routine practice. Second, the presence of HER2-positive CTCs in primary TNBC may be a risk factor for metastatic disease, especially to the brain. Third, the nuclear distribution of DUSP6 in CTCs and brain metastases may be prognostic, the majority of metastatic breast cancer patients with brain metastases had cancer cells positive for DUSP6 and all CTCs examined were also DUSP6 positive. Four, the novel nuclear DUSP6 peptide inhibitor can potentially reduce the risk of brain metastasis. Therefore, DUSP6 may be a therapeutic target to prevent or treat brain metastasis. We also examined DUSP6 expression in surviving tumor cells in a mouse model of breast cancer metastasis treated with Abraxane or anti-PD1. DUSP6-positive cells were enriched in surviving tumor cells, which may indicate a role in resistance to chemotherapy or immunotherapy.

Although nuclear DUSP6 expression was present in CTCs and in the brain metastases of 19 out of 21 patients TNBC patients, this result requires further confirmation in a larger cohort and the samples need to be stratified based on the original hormonal status of the primary tumor in future experiments. Nuclear DUSP6 was not expressed in pleura or lung metastases tissue of TNBC patients, and it remains uncertain whether nuclear-biased DUSP6 in CTCs is exclusively associated with brain metastasis. DUSP6 expression should be analyzed in metastases at other sites as well as obtaining matched CTCs and metastatic lesion tissue samples from different sites to compare for DUSP6 expression. In addition, future studies should examine the nuclear interaction partners of DUSP6 along with the epigenetic machinery that DUSP6 may regulate in cancer biology as well as the underpinning gene signatures associated with DUSP6 in brain metastases. Also, further work should investigate if MAPKs such as ERK1/2 and AKT1/2 are directly mediated by nDUSP6 enriched HER2-postive CTCs. Overall these findings suggest the potential importance of developing nuclear targeting DUSP6 therapeutics for targeting aggressive TNBC cancer spreading, including brain metastasis.

## 4. Materials and Methods 

### 4.1. Patients

Peripheral blood (25 to 40 mL) was collected into 3 or 4 EDTA tubes from five patients with radiologically and histologically confirmed metastatic TNBC before starting a new chemotherapy regimen. Samples were maintained at room temperature and processed within four hours after blood collection. Samples were anonymized, and operators were blinded to clinical outcome. 

### 4.2. Tissues

Tissue from routinely processed archival primary and metastatic breast cancers from the five patients were retrieved from the Department of Anatomical Pathology, ACT Pathology, Canberra Hospital. The tissue diagnosis and receptor status were reviewed by a specialized breast pathologist (JED), who excluded areas of tumor necrosis. 

### 4.3. Animal Studies 

Five-week-old female BALB/c mice were obtained from the Animal Resources Centre (ARC), Perth, and allowed to acclimatize for one week in the containment suites at The John Curtin School of Medical Research (JCSMR). All experimental procedures were performed in accordance with the guidelines and regulations approved by the Australian National University Animal Experimentation Ethics Committee (ANU AEEC). Mice were shaved at the site of inoculation the day before subcutaneous injection with 2 × 10^5^ 4T1 cells in 50 µL PBS into the right mammary gland. Treatment was started on day 12 post inoculation, when tumors reached approximately 50 mm^3^. Tumors were measured using external calipers and volume calculated using a modified ellipsoidal formula ½ (a/b^2^), where a = longest diameter and b = shortest diameter. Mice were treated with the chemotherapy Abraxane (30 mg/kg) and anti-PD1 immunotherapy (10 mg/kg) every 5 days (twice). All treatments were given intraperitoneally in PBS. Tumors were collected on day 27 post inoculation of 4T1 cells for immunofluorescence microscopy. 

### 4.4. Ethics 

All subjects gave their informed consent for inclusion before study participation. The study was conducted in accordance with the Declaration of Helsinki, and the ACT Heath Human Research Ethics Committee (ID code: ETH.11.15.217) approved the protocol on the 4 December 2017.

### 4.5. CTC Enrichment 

CD45-positive cell depletion was performed to enrich CD45-negative expressing cells using the RosettSep CD45 depletion kit (StemCell Technologies, Vancouver, Canada) according to the manufacturer’s instructions and as described previously [5]. 

### 4.6. CTC Detection, Imaging, and Isolation

CD45-negative enriched cells were stained with antibodies targeting human CD45, pan-cytokeratin, and HER2/vimentin/DUSP6 prior to CTC enumeration and sorting by DEPArray V2 or NXT (Silicon Biosystems, Italy). CTCs were defined as nucleated cells lacking CD45 and expressing pan-cytokeratin (CK7, CK8, CK18, and CK19) as described previously [5]. CTCs for mRNA analysis were lysed using the Single Cell Lysis Kit (Thermo Fisher Scientific, Waltham, MA, USA) and stored at –80 °C prior to further analysis. CTCs for immunofluorescence microscopy analysis were cytospun onto coverslips and fixed prior to fluorescence labeling and imaging. 

### 4.7. RNA Isolation, cDNA Synthesis and Pre-Amplification from CTCs

Lysed single CTCs were treated with DNAse I (Thermo Fisher Scientific), and the RNA was converted to cDNA using Superscript VILO (Thermo Fisher Scientific). Pre-amplification was performed using the Nanostring Pre-Amp kit (NanoString Technologies, Seattle, WA). 

### 4.8. Nanostring Analysis

The nCounter XT protocol was used for hybridization, and the data were captured using a Nanostring Sprint instrument (NanoString Technologies). Data analysis was performed using nSolver 4.0 (NanoString Technologies).

### 4.9. ASI Microscopy

For high-throughput microscopy in human tissue samples, protein targets were localized by confocal laser scanning microscopy. Single 0.5 μm sections were obtained using an Olympus-ASI automated microscope with a 60× oil immersion lens running ASI software. The final image was obtained by employing a high-throughput automated stage with ASI spectral capture software. Digital images were analyzed using automated ASI software (Applied Spectral Imaging, Carlsbad, CA) to determine the distribution and intensities automatically with automatic thresholding and background correction of the average nuclear fluorescence intensity (NFI), allowing for the specific targeting of nuclear expression of proteins of interest (in this case DUSP6). 

### 4.10. Nuclear to Cytoplasmic Fluorescence Ratio (Fn/c) Analysis

Digital confocal images were analyzed using Fiji-ImageJ software (Schindelin et al., 2012) to determine the TNFI, TCFI, TFI, or the nuclear to cytoplasmic fluorescence ratio (Fn/c) using the equation: Fn/c = (Fn − Fb)/(Fc − Fb), where Fn is nuclear fluorescence, Fc is cytoplasmic fluorescence, and Fb is background fluorescence. A minimum of *n* = 20 cells were analyzed for each sample set. The Mann–Whitney non-parametric test (GraphPad Prism, GraphPad Software, San Diego, CA) was used to determine significant differences between datasets.

### 4.11. Statistical Analysis 

PRISM-Graph pad was used to calculate the significant differences using the Mann–Whitney Unpaired *t*-test. Significance was plotted as indicated: * *p* < 0.05, ** *p* < 0.01, *** *p* < 0.001, **** *p* < 0.0001 

## 5. Conclusions

We showed for the first time that nuclear-biased DUSP6 was present in CTCs from TNBC patients. Importantly, nuclear DUSP6 was also observed in brain metastases but not lung or pleura metastases from metastatic TNBC patients, while DUSP6 was always cytoplasmic in the primary tumor tissue. These results indicate that a nuclear-biased DUSP6-dependent pathway may be involved in cancer spreading in TNBC patients, including brain metastasis. Inhibition of DUSP6 nuclear translocation reduced the expression of aggressive mesenchymal protein and may be a good candidate therapeutic target to reduce or prevent metastases via CTC in metastatic breast cancer and TNBC patients. Future studies are required to address the precise transcriptional programs underpinning nuclear DUSP6 in cancer spreading in TNBC, especially in brain metastases.

## Supplementary Materials

Supplementary materials can be found at https://www.mdpi.com/1422-0067/20/12/3080/s1.

## Abbreviations

MDPI	Multidisciplinary Digital Publishing Institute
DOAJ	Directory of open access journals
TLA	Three letter acronym
LD	linear dichroism
LSD1	Lysine-specific histone demethylase 1
