# Supplementary material for: Nuclear-Biased DUSP6 Expression is Associated with Cancer Spreading Including Brain Metastasis in Triple-Negative Breast Cancer
Source: Int J Mol Sci. 2019 Jun 24;20(12):3080. doi: 10.3390/ijms20123080 (PMC6627630; doi:10.3390/ijms20123080)
Supplement: Supplementary file 1 [file ijms-20-03080-s001.pdf]

Supplementary Information

Supplementary Figure S1.

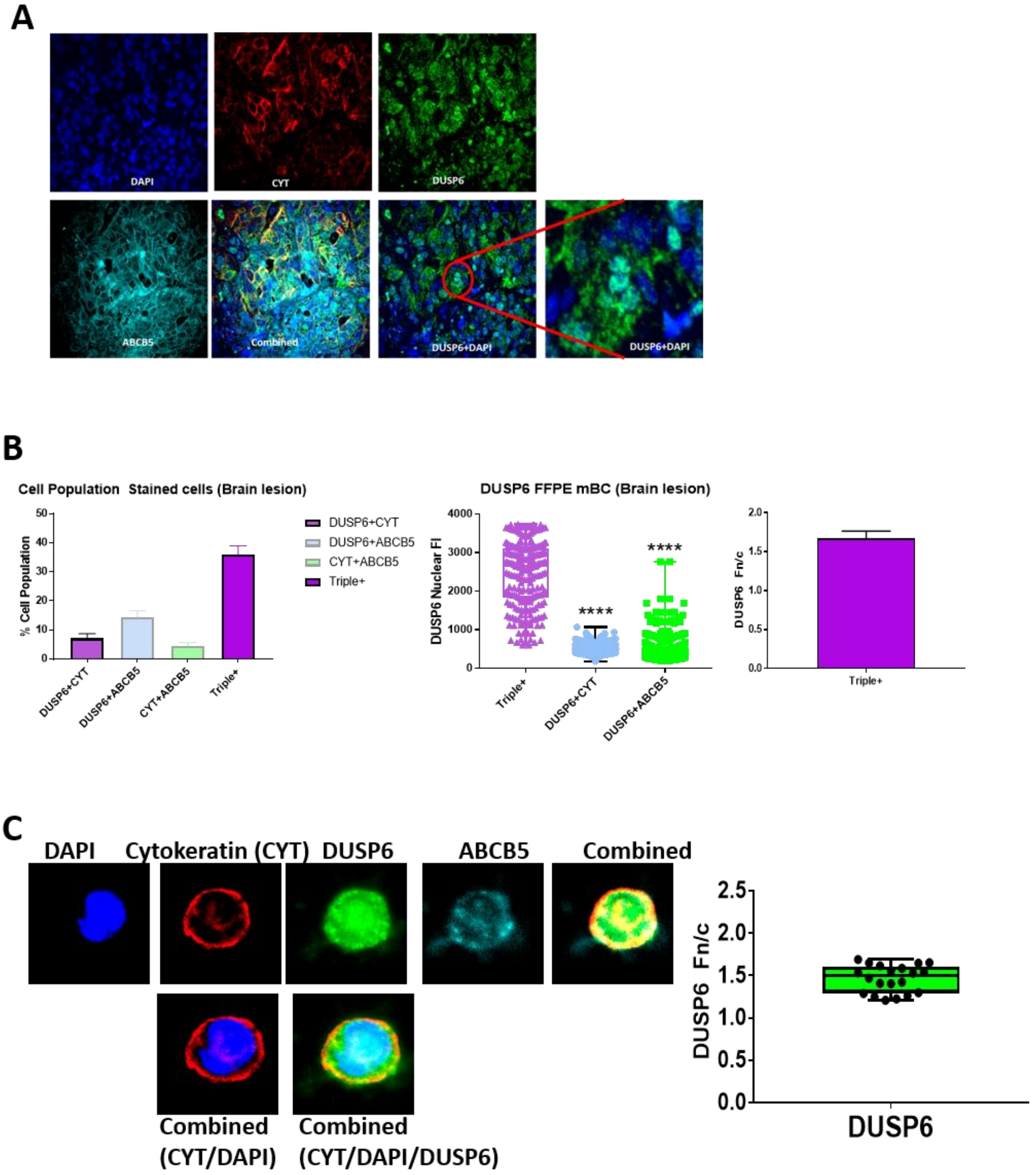

Figure S1. Confirmation of nuclear localization of DUSP6 in brain metastases tissue and CTCs from TNBC patients. (**A**) Brian metastases tissues (**B**)obtained from TNBC patient were labelled with anti-human DUSP6, Cytokeratin (CYT) and DAPI for microscopy. (**B**) percentage of each cell populations and nuclear DUSP6 fluorescence intensity (FI) along with Fn/c were calculated. (**C**) CTCs from TNBC patients were labelled with anti-human DUSP6, CYT and DAPI for microscopy, the Fn/c of DUSP6 was calculated.

Supplementary Figure S2.

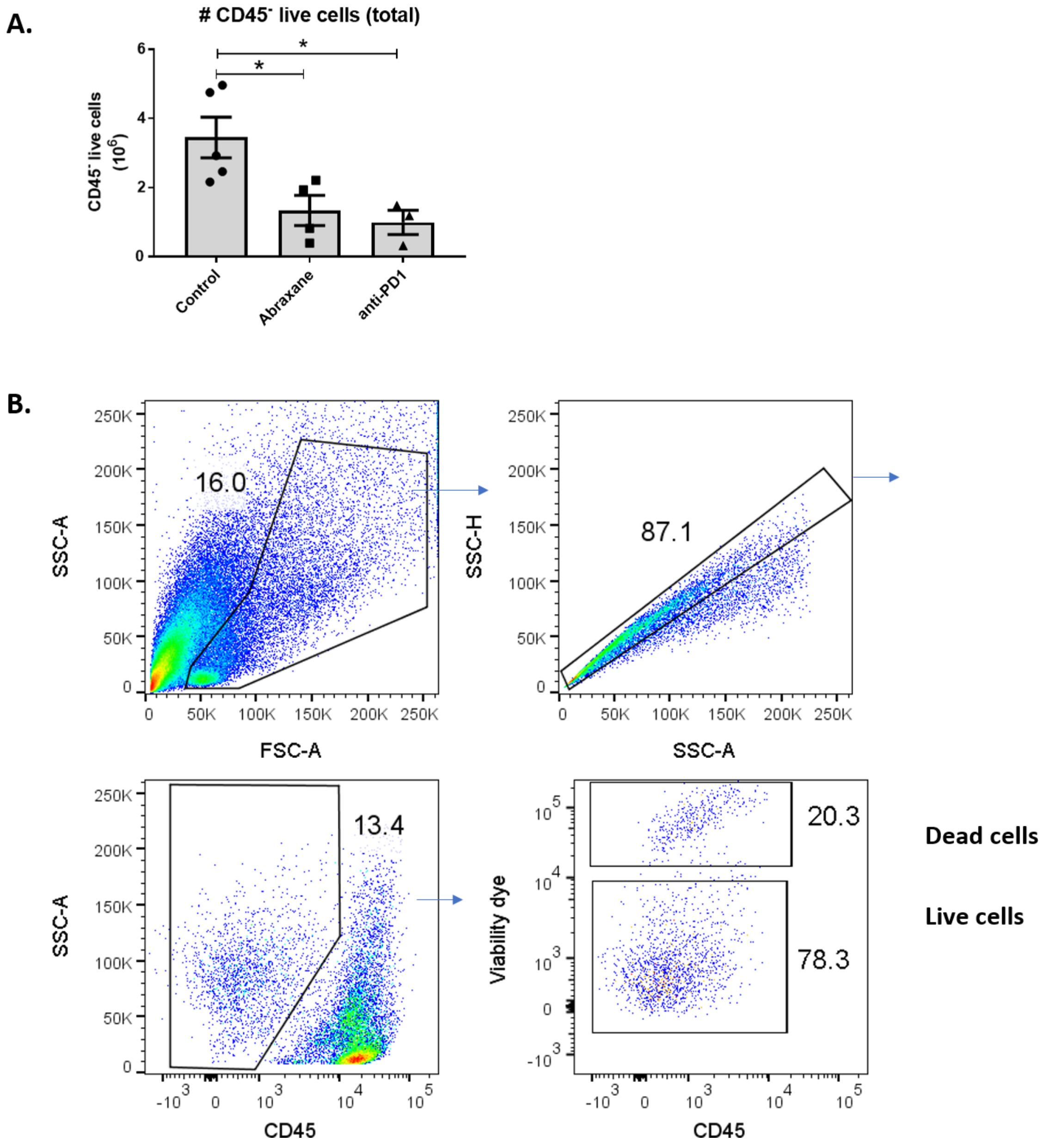

Figure S2. Decreased viable tumor cells post chemotherapy or immunotherapy in 4T1 metastatic mouse cancer model. 4T1 tumors from balb/c mice treated with vehicle control, Abraxane (30 mg/kg) and anti-PD1 (10 mg/kg) were digested into single cell suspension with collagenase IV. Cells were then stained with CD45-Percp Cy5.5 (Biolegend) and BD fixable viability dye 780. Flow cytometry analysis was performed in BD Fortessa. Data was analysed in FlowJo and Prism. Live cells were identified as negative expression of the Viability Dye. (**A**) Graph shows the cell numbers based on the total cell count and percentage of total of live and dead CD45 negative cells (non-immune/non-lymphocyte cells) (n = 3–5 mice). Statistical analysis was performed using the non-parametric Mann-Whitney t-test *$p < 0.05$. (**B**) Gating strategy for data analysis.
